# Multi-Drug and β-Lactam Resistance in *Escherichia coli* and Food-Borne Pathogens from Animals and Food in Portugal, 2014–2019

**DOI:** 10.3390/antibiotics11010090

**Published:** 2022-01-12

**Authors:** Miguel Mendes Costa, Miguel Cardo, Patricia Soares, Maria Cara d’Anjo, Andreia Leite

**Affiliations:** 1 NOVA National School of Public Health, Public Health Research Centre, Universidade NOVA de Lisboa, 1600-560 Lisboa, Portugal; patricia.soares@ensp.unl.pt (P.S.); andreia.leite@ensp.unl.pt (A.L.); 2 Comprehensive Health Research Center (CHRC), Universidade NOVA de Lisboa, 1600-560 Lisboa, Portugal; 3 Veterinary Public Health Department, Directorate-General of Food and Veterinary, 1700-093 Lisbon, Portugal; miguelcardo@dgav.pt (M.C.); andrea.anjo@dgav.pt (M.C.d.); 4 CIISA–Centre for Interdisciplinary Research in Animal Health, Faculty of Veterinary Medicine, University of Lisbon, 1300-477 Lisbon, Portugal

**Keywords:** multi-drug resistance, food-producing animals, zoonotic bacteria, food safety, surveillance

## Abstract

Animal and food sources are seen as a potential transmission pathway of antimicrobial resistance (AMR) to humans. The aim of this study is to describe *Campylobacter*, *Salmonella*, and commensal *Escherichia coli* multi-drug resistance (MDR) in the food chain between 2014 and 2019 in Portugal. AMR surveillance data from food-producing animals and food were assessed. MDR relative frequencies were estimated by bacterial genus and year. AMR profiles were created using observations of resistance to antimicrobial classes from each isolate. Antimicrobial susceptibility testing results were clustered using k-modes. Clusters were described by population, AMR classification, β-lactamases, sample stage, sample type, season, and year. Overall, MDR was more prevalent for *E. coli*, ranging from 74–90% in animal and 94–100% in food samples. MDR was found to be more widespread in resistance profiles that were common among *E. coli* and *Salmonella* isolates and in those exclusively observed for *E. coli*, frequently including (fluoro)quinolones and cephalosporins resistance. β-lactam resistance was observed around 75% to 3rd/4th-generation cephalosporins in *E. coli*. Clusters suggest an escalating MDR behaviour from farm to post-farm stages in all bacteria and that *Salmonella* (fluoro)quinolones resistance may be associated with broilers. These findings support policy and decision making to tackle MDR in farm and post-farm stages.

## 1. Introduction

Anthropogenic, commensal, and environmental bacteria collectively contribute to the antimicrobial resistance (AMR), increasing human vulnerability through resistant strains that colonize the intestinal tract and transfer resistance genes [1,2,3]. In recent decades, antimicrobial-resistant bacteria have been recognized as a public health threat that has led to an increase in the global burden of infectious disease with more than 670,000 infections and 33,000 deaths in the EU and 700,000 deaths worldwide each year due to treatment failure [4,5,6].

AMR evolution can be influenced jointly by factors, such as bacteria environmental persistence, host immune status, microflora composition, and antimicrobial interventions [7]. This evolution can result in multi-drug resistance (MDR), i.e., resistance to three or more antimicrobial classes, affecting the usefulness of multiple last-resort antimicrobials, such as 3rd-generation cephalosporins and carbapenems, two of the leading antimicrobial classes used in the treatment of MDR infections [8,9]. Furthermore, it may enhance the need to use other last-resort antimicrobials [10]. While AMR cannot be realistically eradicated, antimicrobials will continue to lose their efficacy, and, in the near future, more people may die from infections as treatment options disappear [11].

Resistance to high-priority antimicrobials to human medicine has been given considerable attention with regard to food-producing animals due to their potential role in transferring, directly or via food, resistant bacteria to humans [12]. Furthermore, *Escherichia coli*-combined resistance to multiple last-resort antimicrobials, such as polymyxins (PLM) and 3rd/4th-generation cephalosporins (C3G/C4G), is becoming a more common source of MDR due to extended spectrum ß-lactamases (ESBLs) [4]. In the EU, MDR levels between 2018 and 2019 were observed in more than one third of *Salmonella* isolates from broiler and pig samples, and in more than two thirds in *E. coli* from pigs, broilers, and turkeys, as well as derived carcase samples in some member states [13]. In Portugal, notable MDR has already been reported in *Salmonella* isolates from pigs (55%) and pork products (61%) in 2013, *E. coli* from poultry carcasses (56%) in 2016, and *Enterobacteriaceae* from livestock manure from poultry (71%) and pig (79%) farms in 2016 and 2017. MDR was distributed between three and five antimicrobials including tetracyclines (TET), sulphonamides (SLP), chloramphenicol (CHL), amoxicillin, and/or trimethoprim (TMP) [14,15,16]. Nevertheless, surveillance evidence is needed regarding MDR behaviour and how it escalates across the food production stages in Portugal to support measures in each stage that may effectively tackle this public health concern and minimize its impact on animal and human health.

This study assesses MDR in animal farm and post-farm stages of the Portuguese food system to provide insights on the potential impact of AMR from animal sources. Our objectives were, thus, to describe MDR prevalence and trends, and the unique and shared AMR profiles from *Campylobacter*, *Salmonella,* and *Escherichia coli* from poultry and pig populations and derived food products between 2014 and 2019 in Portugal. Furthermore, we described MDR clusters according to surveillance variables, describing the characteristics of the samples.

## 2. Results

**Figure 1:**
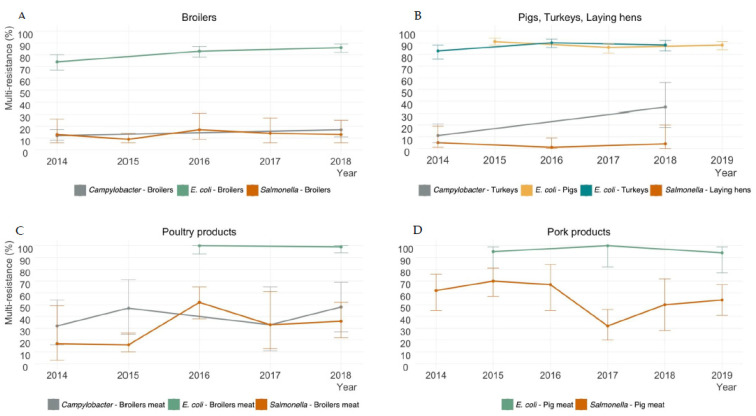
Multi-resistance in bacterial isolates collected from animal populations (**A**,**B**) and food products (**C**,**D**), 2014–2019.

Overall, 2524 commensal *E. coli*, 464 *Salmonella,* and 458 *Campylobacter* isolates were assessed from food-producing animal samples, as well as 253 commensal *E. coli*, 455 *Salmonella,* and 83 *Campylobacter* isolates from animal-derived food products, from 2014 to 2019. Multi-resistance prevalence across all surveillance years for *Salmonella*, *E. coli*, and *Campylobacter* is presented in Figure 1 for food-producing animals (A—broilers; and B—turkeys, laying hens, and pigs) and food products (C—broilers meat; and D—pigs meat). Multi-resistance was more prevalent for *E. coli* isolates, ranging from 74% (95% CI: 67–80%) to 90% (95% CI: 86–93%) in animal populations (Figure 1A,B) and 94% (95% CI: 77–99%) to 100% (95% CI: 93–100%) in food products (Figure 1C,D). *Campylobacter* isolates demonstrated an MDR increase in 2018 to 35% (95% CI: 18–56%) in turkey (24 percentage points (pp)) (Figure 1B), 17% (95% CI: 11–25%) in broilers (5 pp) (Figure 1A), and 48% (95% CI: 27–69%) in broiler products (16 pp) (Figure 1C), compared to 2014. From 2017 to 2019, MDR in *Salmonella* isolates from pork products revealed an increase to 54% (95% CI: 41–67%) (22 pp), but less when compared to 2014 (8 pp) (Figure 1D). From 2014 to 2018, *Salmonella* MDR increased to 36% (95% CI: 22–52%) in broiler products (19 pp) (Figure 1C). According to the definitions considered and antimicrobials tested in the epidemiological panel, no isolate was found to be extensively drug-resistant or pan drug-resistant.

**Figure 2:**
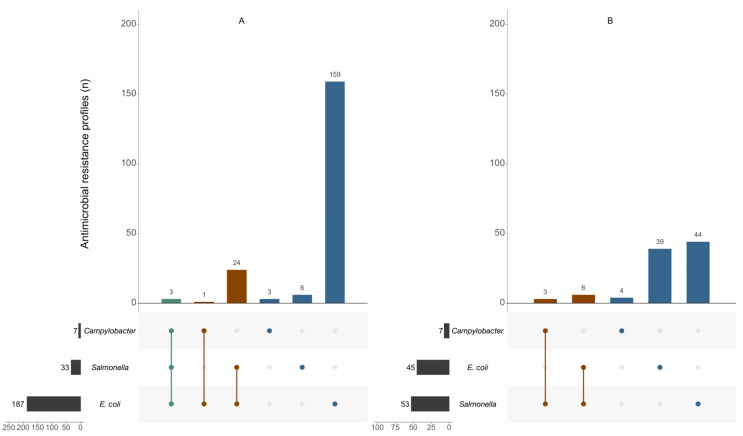
AMR profiles within food-producing animal populations (**A**) and food products (**B**) from *Campylobacter*, *Salmonella,* and *E. coli* isolates, 2014–2019. Dots represent the type of bacteria and connecting lines indicate that profiles are common between bacteria. Colours represent shared profiles between bacteria, green—all types of bacteria; red—two types of bacteria, and blue—one type of bacteria, representing unique profiles. The number of distinct AMR profiles identified for each bacteria are presented in horizontal bar graphs displayed on the left.

AMR profiles distribution for bacterial isolates from food-producing animal samples and food products are presented in Figure 2, demonstrating unique and shared AMR profiles in the bacterial genera studied. The highest number of unique AMR profiles was observed in *E. coli* isolates from food-producing animal samples and in *Salmonella* isolates from food products (respectively, 159 and 44 different profiles). Shared profiles were mostly observed between *Salmonella* and *E. coli* isolates, 24 different profiles were observed in isolates from animal samples, and 6 profiles were observed in isolates from food products. The three most observed unique or shared AMR profiles for *E. coli*, *Salmonella*, and *Campylobacter* are presented in Table 1. For isolates from food-producing animal samples (*n* = 3216), the most observed profiles were TET-fluoroquinolones and quinolones (F(Q)/(fluoro)quinolones)-C3G-CHL-SLP- Penicillins (PEN) in *E. coli* (124 of 1462 isolates), Macrolides (MAC)-TET-F(Q) in *Campylobacter* (57 of 67 isolates), and resistance only to CHL in *Salmonella* (3 of 10 isolates). As for shared profiles, TET-F(Q)-CHL-SLP-PEN-TMP was the most observed between *E. coli* and *Salmonella* (189 of 947 isolates), MAC-F(Q) between *E. coli* and *Campylobacter* (3 of 3 isolates), and TET-F(Q) between the three kinds of bacteria tested (354 of 727 isolates). For isolates from food products (*n* = 690), unique AMR profiles were TET-F(Q)-C3G-SLP-PEN-TMP for *E. coli* (35 of 181 isolates), TET-SLP-PEN for *Salmonella* (69 of 232 isolates), and MAC-TET-FQ for *Campylobacter* (26 of 57 isolates). Most observed shared profiles in isolates from food products were TET-F(Q)-C3G-CHL-SLP-PEN-TMP between *E. coli* and *Salmonella* (49 of 92 isolates) and F(Q) between *E. coli* and *Campylobacter* (59 of 128 isolates).

Antimicrobial susceptibility testing (AST) results were used to offer an overview of the datasets and identify distinct clusters in each type of bacteria while assessing similarity between isolates and number of mismatches in AST results. *E. coli, Salmonella,* and *Campylobacter* clustering results are reported in Table 2, and additional characterization of each cluster using surveillance variables is available in Tables S1–S3. All antimicrobial susceptibility results, both sensitive and resistant antimicrobial observations, were used for clustering. Subsequently, clusters were characterized using surveillance variables using data from samples and bacterial isolates that could offer insights on MDR occurrence. The number of optimal clusters for *E. coli* was four (Figure S1), and purity of clusters was 0.79. Cluster 1 (*n* = 1292) presented high AMR to PEN (92%), CHL (73%), SLP (96%), TMP (89%), F(Q) (73%), and TET (91%), with more than half of the isolates showing MDR 5–6 (52%). Cluster 2 (*n* = 535) had isolates susceptible against most tested antimicrobials, except F(Q) (39%), TET (59%), and PEN (57%), and around one third of isolates revealed MDR 3–4 (36%). Cluster 3 (*n* = 799) displayed high AMR to PEN (97%), SLP (82%), F(Q) (81%), TET (79%), and C3G (92%) with frequent MDR 5–6 (64%). Critically important AMR was mainly observed in cluster 4 (*n* = 151) to F(Q) (88.7%), C3G (100%), MAC (46%), and PLM (45.7%), but also to PEN (100%), AMN (100%), CHL (83%), SLP (96%), TMP (92%), and TET (95%), with most isolates displaying MDR ≥7 (93%). Most ESBL/AmpC-producing *E. coli* were observed in clusters 3 and 4 (respectively, 90% and 97%). Broilers were the leading population in cluster 3 (32%) and pigs in other clusters (around 39%). Most samples were collected in slaughterhouses, but those from retail (meat samples) were mostly grouped with clusters 3 and 4. Approximately half of the isolates from 2018 were grouped with cluster 3 and 4. Other clusters revealed a consistent distribution of isolates across all surveillance years. Most of the isolates were collected during autumn.

Resistance profiles of ESBL/AmpC-producing *E. coli* were observed to both C3G and C4G in most isolates from animal and food samples (respectively, 77%; *n* = 846; and 74%; *n* = 188). β-lactam AMR frequencies and resistance profiles observed in *E. coli* isolates that were further submitted to phenotypical characterization and tested for ESBL/AmpC are in Table S4.

The number of optimal clusters for *Salmonella* was four (Figure S2), and the purity of clusters was 0.97. Cluster 1 (*n* = 141) displayed resistance in more than half isolates to TET (97%), PEN (92%), and SLP (84%). Cluster 2 (*n* = 619) displayed overall susceptibility to tested antimicrobials, except F(Q) (44%). Cluster 3 (*n* = 30) also displayed overall susceptibility to tested antimicrobials, except TET (100%). Cluster 4 (*n* = 129) presented resistance mostly to TET (78%), PEN (78%), SLP (99%), TMP (100%), and in more than one third to F(Q) (42%) and CHL (39%). Cluster 1 mainly included isolates from pork products (67%) and processing plants (62%), during autumn (39%), 2015 (24%), and 2016 (25%), and displayed MDR mostly to three and four antimicrobials (75%). Cluster 2 had most isolates from broiler samples (animal/environmental) collected in farms (66%), mostly in 2015 (48%) with summer (33%) and winter being the seasons with more predominant sampling and displaying a high number of isolates with susceptibility to all antimicrobials (51%) and mono-resistance (42%). Cluster 3 included mostly pig samples (80%) collected in slaughterhouses (33%) and processing plants (50%), displaying overall mono-resistance (93%). Cluster 4 also included pork products as the most prevalent (48%) with significant sampling during summer (36%) and in 2015 (36%), presenting MDR to 3–4 (49%) and 5–6 (39%) antimicrobials.

For *Campylobacter*, three clusters were identified (Figure S3) and the purity of clusters was 1. Cluster 1 (*n* = 479) displayed high AMR to AMN (96%), F(Q) (98%), and TET (100%), with most profiles showing dual resistance (77%). Samples were mainly collected in slaughterhouses (84%), during 2014 (60%) and 2018 (33%), most in autumn (52%), and of broiler origin (66%). Cluster 2 (*n* = 11) presented susceptibility to all tested antimicrobials, most from broiler samples (73%). As for cluster 3 (*n* = 51), AMR was mostly observed to F(Q) (100%) and AMN (98%) in caecal samples collected in slaughterhouses (87%), mainly in 2014 (84%) during summer (45%), and was generally mono-resistant (94%).

## 3. Discussion

In this study, a high diversity of MDR profiles was observed for *E. coli,* including HP-CIAs resistance to F(Q), C3G, MAC, and PLM, especially in pig and turkey caecal samples. Overall, MDR from five to six antimicrobials was observed in most animal and food isolates. Resistance to seven or more antimicrobials was observed in isolates from both animal and food samples. In line with our observations, other studies have reported high MDR levels in *E. coli* through the food chain [13,17,18,19,20]. Overall, *E. coli* MDR levels were reported at 76% for pig production in South Africa; 75% on farms and 50% in slaughterhouses; 72% from pig and chicken samples in Malaysia; 78% in broiler products from retail in Bangladesh with AMR above 80% to PEN, TET, and MAC; and 92% from chicken samples in Nigeria with resistance in more than two thirds to AMN, F(Q), PEN, TET, and SLP/TMP [17,18,19,20]. *E. coli* MDR was observed to differ considerably across EU member states, ranging from 3% to 85% in pigs, 0% to 87% in broilers, and 0% to 78% in turkeys. MDR patterns amongst pigs and broilers were fairly similar in the EU, revealing TET, PEN, SLP, and TMP as the most frequent in both animal populations, while F(Q) resistance was further observed in poultry populations [13].

ESBL/AmpC-producing *E. coli* (ESBL/pAmpC *E. coli*) were observed in all *E. coli* from broiler products. β-lactam resistance was mostly observed to C3G and C4G, and less than one fifth of the isolates were tested with other antimicrobials. These ESBL/pAmpC *E. coli* observations are in accordance with those from other studies [21,22,23,24]. ESBL/pAmpC bacteria are commonly multi-resistant and their origin has been linked with the use of extended spectrum cephalosporins in animals and co-selection from other antimicrobials [25]. The bacteria ability to hydrolyse ß-lactam antimicrobials may provide them with an opportunity to become a persistent source of infection and escalate the overuse of last-resort antimicrobials in human medicine [26,27,28]. Furthermore, ESBL carriage from animals to humans associated with working/living on farms has already been reported with ESBL-producing *E. coli* carrying pigs [29]. Cross-contamination from other products, environment, equipment, and workers’ handling in different production and retail units can also enhance the development of ESBL/pAmpC bacteria (Kaesbohrer et al., 2019). Additionally, transmission of ESBL/pAmpC *E. coli* to humans through the food chain can occur if the exposure implies the consumption of raw meat products [23]. Consequently, a better understanding of bacterial ecology, diversity, and population dynamics could play a role in the elaboration of new preventive measures to support good hygiene practices aimed at eliminating resident bacterial flora and reducing carcasses contamination in food processing environments, probably adapted for different food production steps (e.g., evisceration, splitting of carcasses) [30]. These preventive strategies may be supported by both surveillance and monitoring of slaughter and processing food environments; this involves targeting critical contamination and biofilms formation points to identify opportunities that could help tackle MDR and β-lactamases occurrence.

MDR to three or four antimicrobials occurred frequently in *Salmonella* isolates assembled to clusters 1 and 4, mostly pork isolates. Increasing MDR trends were observed for both broiler and pork products during the surveillance years assessed in Portugal. High *Salmonella* MDR levels were previously reported between 2018 and 2019 from chicken (81%) and pork (73%) samples collected at retail in China [31], broiler (81%) farm samples in Malaysia [32] as well as other EU member states besides Portugal, broiler (33%) and pig (43%) farm samples, and broiler carcases in Austria (87%) and in Slovenia (91%) [13]. Our findings indicate an emergence of *Salmonella* MDR mostly to TET, PEN, SLP, F(Q), or TMP in isolates collected from food samples, as well as a concern with mono-resistance to F(Q) at the farm level for broilers. These results suggest the need to act regarding F(Q) use at the farm stage in broilers production, but also to direct appropriate post-farm interventions. Furthermore, surveillance and data collection are needed concerning other MDR determinants (e.g., resident bacterial flora of food production environments, and disinfectants used for hygiene practices in each environment) that can play a role in their diversity and evolution.

A predominance of *Campylobacter* isolates with at least F(Q) resistance was observed in almost all poultry isolates, mainly in broilers. MDR profiles were observed for all antimicrobial classes tested. A study conducted in Poland has reported similar resistance levels to F(Q) in turkey and broiler *Campylobacter* strains (100% in both populations) [33]. *Campylobacteriosis* human cases are usually treated with F(Q) or MAC and *Campylobacter* AMR trends to these antimicrobials have been increasing in the European region. Thus, this might instigate treatment failures and the use of other antimicrobials [34,35]. In this sense, *Campylobacteriosis* human cases can become more demanding to treat and start limiting treatment options for animals as they become dependent on viable human medicine options.

AMR profiles differed mostly between *Salmonella* isolates from food-producing animal samples and those from food products. This example of *Salmonella* indicates a wider resistance profile in food products than compared to animal samples, probably due to determinants of resistance to antimicrobials within post-farm stages. Bacteria can acquire the ability to tolerate antimicrobials through different types of selective pressure, such as contact with antibiotics, heavy metals, and biocides, or persistent colonization of food-processing environments with resistant bacterial strains. The adaptative reaction can result in cross-resistance or co-selection of resistance, and can be a determinant of acquired resistance to unrelated and clinically relevant antimicrobials [36]. Consequently, MDR prevention in Portugal may need to improve both critical control points HACCP-based procedures, biosecurity, and farming measures by refining the knowledge and filling in data gaps on microbial environments and their dynamics in different food production stages.

The cluster analyses offer an overview of the AMR surveillance dataset, and enable us to identify distinct bacterial resistance similarities and discrepancies and detect which characteristics may support a better understanding of MDR development in the food chain. The characterization of these clusters provided insights on how antimicrobial susceptibility clusters were associated with surveillance data from the samples collected and bacterial isolates, supporting decision making for preventive strategies in each food production stage. Nevertheless, some limitations need to be considered: (i) laying hens and other populations/food categories were excluded, due either to a heterogeneous distribution of the susceptibility testing results with a pan-susceptibility predominance in the isolates tested or a small sample size; (ii) *Campylobacter* isolates were tested to only six antimicrobials representing four antimicrobial classes, limiting comparisons concerning clusters and classifications; (iii) a small number of isolates by bacterial genus in some years (e.g., a small sample size of *Salmonella* (fewer than 100 isolates) in 2019 as well as for *Campylobacter* in most surveillance years); and (iv) a large difference in the number of isolates for animal populations and food products may have had an influence in our conclusions from the diversity of AMR profiles observed. These limitations should be seen as targets for improving the existing AMR surveillance. Furthermore, it would be interesting to include human and environmental AMR data in our models and further assess similarities and discrepancies from samples of different sectors that may play a direct or indirect role in the emergence and selection of MDR bacteria. This could be attempted using a One Health concept that looks at systems that are not completely compatible with a single conceptualization of health, directing attention instead toward shared physiological processes and common susceptibilities to pathogens that lead to adverse health-related outcomes [37]. This concept was introduced at the beginning of this century, recognizing that human and animal health are interdependent, coexist, and evolve in ecosystems in which they interact with other living beings, such as plants and microorganisms [38]. In this sense, an infectious risk, such as AMR, should be addressed in a multi-sectorial approach to explore the ecological role of antimicrobials and their resistance genes, infectious agents’ proliferation, and changes within hosts microbiota and evolution of pathogenic traits [39].

Overall, this study offers insights on MDR occurrence on food-producing animals and food products, indicating that β-lactam resistance may be associated with multi-resistance in farm and post-farm stages, especially for *E. coli*. New regulations, electronic prescribing systems, and restrictions on antimicrobials used in veterinary medicine must be put into action to safeguard treatment options and antimicrobials efficacy in human medicine. Our results give valuable support to policy and decision makers to tackle important MDR profiles and the escalating behaviour of multi-resistance in farm and post-farm stages. Further research should include other sectors that can impact the emergence and selection of AMR to improve knowledge and develop alternatives that will protect public health.

## 4. Material and Methods

### 4.1. Study Design, Setting, and Data Collection

Bacterial isolates acquired from food-producing animals (broilers, laying hens, turkeys, and pigs) and food products (broiler and pork products) were tested for AMR phenotypical analysis (profiles and trends). The dataset used derives from the yearly surveillance programme to monitor AMR in commensal *Escherichia coli, Salmonella,* and *Campylobacter*, conducted by the Portuguese Authority, the Directorate-General for Food and Veterinary and led in accordance with the European Commission decision 2013/652/EU (2013) and Directive 2003/99/EC (2003) for technical specifications on randomized sampling, monitoring, and reporting of antimicrobial resistance in zoonotic and commensal bacteria [40,41]. Poultry and derived food products were collected from farms, slaughterhouses, processing plants, and retail outlets in all surveillance years. Pig and derived food products were collected from slaughterhouses, processing plants, and retail outlets in 2015, 2017, and 2019. Prospective and retrospective sampling strategies were used. A prospective sampling was applied in slaughterhouses (minimum of 60% of the animal production output in the prior year) for caecal content and in retail outlets for meat samples. Chilled fresh meat samples were collected at retail level (minimum of 80% of the Portuguese population), consistent with NUTS-3 areas (Nomenclature of Territorial Units for Statistics—level III). Samples were complemented by food business operators when sample representativeness was insufficient during official sampling at the retail level. Food business operators’ sampling was conducted over a stratified sampling plan in slaughterhouses and processing plants with allocation proportional to the size of the isolate collections available in laboratories. Retrospective sampling was implemented to obtain *Salmonella* isolates from: (i) *Salmonella* control programmes covering environmental and faecal samples at farm level; and (ii) laboratory isolates from a *Salmonella* surveillance programme, complemented with those from food business operators’ own checks for hygiene control to assess *Salmonella* trends and microbiological risks in poultry and pig carcasses from slaughterhouses (randomly selected) [42,43].

### 4.2. Microbiology Surveillance Data

Non-clinical bacterial isolates were submitted to AST by the national official laboratory, the National Institute for Agrarian and Veterinary Research, in line with EUCAST guidelines, using harmonized epidemiological cut-offs; valid dilution ranges; antimicrobial discs with specific concentrations (mg/L); and minimum inhibitory concentrations regarding the following antimicrobials: ampicillin, temocillin, azithromycin, erythromycin, sulfamethoxazole, tetracycline, tigecycline, gentamicin, streptomycin, cefoxitin, cefotaxime, ceftazidime, cefepime, colistin, ciprofloxacin, nalidixic acid, meropenem, imipenem, ertapenem, trimethoprim, and chloramphenicol [44,45].

Bacterial isolates were first submitted to an epidemiological AST panel. *Salmonella* and *Escherichia coli* isolates were tested to PEN, MAC, SLP, TET, AMN, C3G, PLM, F(Q), CBP, TMP, and CHL. *Campylobacter* isolates were tested to MAC, AMN, TET, and F(Q). Erythromycin and streptomycin were tested only in *Campylobacter* isolates. For *Salmonella* and *E. coli*, only those isolates resistant to C3G and/or CBP were further tested for phenotypic characterization of β-lactam AMR to 2nd- and 4th-generation cephalosporins (C2G/4G), β-lactamase-resistant PEN (temocillin), other CBP (imipenem and ertapenem), antimicrobial combinations of clavulanic acid with C3G (cefotaxime or ceftazidime), and bacterial synergy with extended spectrum beta-lactamases (ESBLs) and other cephalosporinases (AmpC). Other β-lactam antimicrobials, already tested in the epidemiological panel, were included (cefotaxime, ceftazidime, and meropenem). ESBLs/AmpC refers to isolates with ESBLs and/or an AmpC phenotype. ESBLs isolates are those resistant to cefotaxime and/or ceftazidime, a synergy test positive for any of these antimicrobials in combination with clavulanic acid, and meropenem and cefoxitin susceptibility. AmpC-producing bacteria are those resistant to cefoxitin together with cefotaxime and/or ceftazidime, both synergy tests negative and meropenem susceptibility. ESBLs and AmpC isolates are those resistant to cefoxitin together with cefotaxime and/or ceftazidime, a synergy test positive and meropenem susceptibility [46].

### 4.3. Study Variables

AMR results were recoded for each antimicrobial, and the isolate was deemed as resistant or susceptible to a certain antimicrobial. Intermediate results with a minimum inhibitory concentration matching the cut-off value were considered susceptible [45]. Surveillance variables were: year (2014, 2015, 2016, 2017, 2018, and 2019), population/food categories (broilers, turkeys, pigs, broiler products, and pork products), seasons (spring, summer, autumn, and winter), ESBLs/AmpC presence (positive or negative), sample stage (farm, slaughterhouse, processing plant, or retail), and sample type (animal, including faecal or caecal samples; environmental, including boot swabs and dust; and food, corresponding to meat samples). All the above variables were created by aggregating category data that had common characteristics (e.g., the categories “*Gallus gallus* (fowl) broilers before slaughter” and “*Gallus gallus* broilers during rearing period” were aggregated and named as “broilers” for population/food categories). A variable for resistance classification based on Magiorakos et al. (2011) [9] was created and included the following levels: pan-susceptibility to tested antimicrobials, mono-resistance (resistance to one antimicrobial class), dual resistance (resistance to two antimicrobial classes), MDR to three and/or four antimicrobial classes, MDR to five and/or six antimicrobial classes, and MDR to seven or more antimicrobial classes (except for *Campylobacter* isolates, which were tested to only four antimicrobial classes).

### 4.4. Statistical Analysis

Multi-resistance relative frequencies and 95% confidence intervals (95% CI) were estimated by bacterial genus and surveillance year. AMR profiles (i.e., sequences of antimicrobial resistance results to tested antimicrobial classes in each isolate) were created using observations of resistance to antimicrobial classes from each bacterial isolate. Unique and shared AMR profiles between bacterial genus were assessed for food-producing animal populations and food products by comparing AMR profiles from aggregated AST results of each *Salmonella*, *Campylobacter,* and commensal *E. coli* isolates. Additionally, all AST results, including those that were susceptible, were clustered by an extension of K-means, i.e., the *k*-modes algorithm, to identify distinct AMR clusters in each bacteria. This method uses simple-matching distance to ascertain the dissimilarity of two isolates, measuring the number of mismatches from AMR observations. Modes were used for clusters and AMR results were separated into *k* groups. The similarity between two isolates differed according to the number of mismatches in AMR observations (e.g., a reduced number of mismatches resulted in increased similarity between isolates) [47]. Samples of turkey meat and laying hens were excluded from the *Salmonella* k-modes model, due to the low number of samples (fewer than 30) and high number of isolates susceptible to antimicrobials, respectively, which affected the clustering analysis. Pork products were excluded from the *Campylobacter* k-modes model due to low number of samples (fewer than 30). The elbow method was used to determine the number of optimal clusters to use in the K-modes. The purity of cluster was calculated to assess the external validity of the clustering results. Clusters were further characterized according to surveillance variables and resistance classification using absolute and relative frequencies within each cluster. Data analysis was conducted using R 4.0.3 [48] with *Klar* packages [49] and UpSetR packages [50].

## Figures and Tables

**Table 1:** Unique and shared profiles between bacteria in food-producing animals and derived food products, 2014–2019.

		Unique Profiles			Shared Profiles		
		*E. coli* *n =* 1462	*Salmonella* *n =* 10	*Campylobacter* *n =* 67	* E. coli *Salmonella* *n =* 947	* E. coli *Campylobacter* *n = 3*	* E. coli *Salmonella* *Campylobacter* *n =* 727
Food-producing animals *n =* 3216	1	TET-FQ-C3G-CHL-SLP-PEN (124)	CHL (3)	MAC-TET-FQ (57)	TET-FQ-CHL-SLP-PEN-TMP (189)	MAC-FQ (3)	TET-FQ (354)
	2	TET-FQ-C3G-SLP-PEN-TMP (84)	FQ-AMN (2)	TET-FQ-AMN (9)	TET-FQ-C3G-CHL-SLP-PEN-TMP (186)	-	FQ (301)
	3	TET-FQ-PEN (76)	MAC (2)	MAC-FQ-AMN (1)	TET-FQ-SLP-PEN-TMP (133)	-	TET (72)
		*E. coli* *n =* 181	*Salmonella* *n = 232*	*Campylobacter* *n =* 57	* E. coli *Salmonella* *n =* 92	* Campylobacter *Salmonella* *n =* 128	* All three bacteria *n =* 0
Food products *n =* 690	1	TET-FQ-C3G-SLP-PEN-TMP (35)	TET-SLP-PEN (69)	MAC-TET-FQ (26)	TET-FQ-C3G-CHL-SLP-PEN-TMP (49)	FQ (59)	-
	2	TET-FQ-C3G-CHL-SLP-PEN (27)	TET-CHL-SLP-PEN-TMP (13)	MAC-TET-FQ-AMN (15)	TET-C3G-SLP-PEN-TMP (11)	TET-FQ (43)	-
	3	FQ-C3G-PEN (21)	MAC-TET-SLP-TMP (12)	TET-FQ-AMN (14)	TET-FQ-C3G-SLP-PEN (11)	TET (26)	-

* Combined number of bacterial isolates is presented for each AMR profile shared between types of tested bacteria. AMN—aminoglycosides; MAC—macrolides; F(Q)—fluoroquinolones and quinolones; TET—tetracyclines; CHL—chloramphenicol; PLM—polymyxins; PEN—penicillins; SLP—sulphonamides; TMP—trimethoprim; C3G—3^rd^-generation cephalosporins; CARB—carbapenems.

**Table 2:** Clusters using antimicrobial susceptibility testing results from *Escherichia coli*, *Salmonella,* and *Campylobacter* isolates observed between 2014–2019 in food-producing animals and derived meat samples. Clusters were created by assessing similarity between isolates and number of mismatches in AST results. Resistance percentages by antimicrobial class and bacterial genus are presented for each cluster.

	Clusters	AMN (%)	MAC (%)	F(Q) (%)	TET (%)	CHL (%)	PLM (%)	PEN (%)	SLP (%)	TMP (%)	C3G (%)	CARB (%)
*E. coli*	1 *n =* 1292	9.0	14.6	73.4	91.3	73.3	15.0	92.0	95.7	88.7	28.9	1.2
*n =* 2777	2 *n =* 535	3.9	3.4	39.1	58.5	2.6	4.1	57.2	8.0	5.0	16.6	1.1
	3 *n =* 799	3.9	12.9	81.1	79.2	26.4	7.8	97.4	82.0	32.2	91.9	0.3
	4 *n =* 151	100.0	45.7	88.7	94.7	82.8	32.5	100.0	96.0	92.1	100.0	0.0
*Salmonella*	1 *n =* 141	6.4	2.1	12.8	97.2	14.9	5.7	92.2	84.4	0.0	2.1	
*n =* 919	2 *n =* 619	2.3	1.3	43.8	0.0	0.6	5.0	6.8	1.3	1.5	0.3	
	3 *n =* 30	0.0	0.0	6.7	100.0	0.0	0.0	0.0	0.0	0.0	0.0	
	4 *n =* 129	5.4	24.8	41.9	77.5	38.8	3.9	77.5	99.2	100.0	14.0	
*Campylobacter*	1 *n =* 479	96.5	18.2	97.5	100.0							
*n =* 541	2 *n =* 11	0.0	0.0	0.0	0.0							
	3 *n =* 51	98.0	5.9	100.0	0.0							

## Data Availability

No new data were created or analysed in this study. Data sharing is not applicable in this study.

## Supplementary Materials

The following are available online at https://www.mdpi.com/article/10.3390/antibiotics11010090/s1, Figure S1: Number of optimal clusters for *E. coli* clustering model using the elbow method, Figure S2: Number of optimal clusters for *Salmonella* clustering model using the elbow method, Figure S3 Number of optimal clusters for *Campylobacter* clustering model using the Elbow method, Table S1: Surveillance variables and resistance classification associated with clusters from *Escherichia coli* antimicrobial susceptibility testing results, Table S2: Surveillance variables and resistance classification associated with clusters from *Salmonella* antimicrobial susceptibility testing results, Table S3: Surveillance variables and resistance classification associated with clusters from *Campylobacter* antimicrobial susceptibility testing results, Table S4: *Escherichia coli* β-lactam antimicrobial resistance and most frequent profiles in animal and food samples tested for ESBL/AmpC.
