# Supplementary material for: Multi-Drug and β-Lactam Resistance in Escherichia coli and Food-Borne Pathogens from Animals and Food in Portugal, 2014–2019
Source: Antibiotics (Basel). 2022 Jan 12;11(1):90. doi: 10.3390/antibiotics11010090 (PMC8773433; doi:10.3390/antibiotics11010090)
Supplement: Supplementary file 1 [file antibiotics-11-00090-s001.zip › antibiotics-1501724-supplementary.pdf]

Table S1: Surveillance variables and resistance classification associated with clusters from *Escherichia coli* antimicrobial susceptibility testing results.

			Cluster 1 *n*= 1292		Cluster 2 *n*= 535		Cluster 3 *n*= 799		Cluster 4 *n*= 151	
		**N**	**%**	**n**	**%**	**n**	**%**	**n**	**%**	**n**
**Population/ food**	**Broilers**	821	26.5	342	34.4	184	31.5	252	28.5	43
	**Turkeys**	694	31.3	405	26.4	141	16.0	128	13.2	20
	**Pigs**	1009	38.1	492	36.8	197	32.	258	41.1	62
	**Pork products**	93	1.5	19	1.9	10	7.1	57	4.6	7
	**Broiler products**	160	2.6	34	0.6	3	13.0	104	12.6	19
**Resistance**	**Pan-S**	67	0.0	0	12.5	67	0.0	0	0.0	0
	**Mono**	118	0.0	0	22.1	118	0.0	0	0.0	0
	**Dual**	218	0.9	11	28.8	154	0.6	53	0.0	0
	**MDR (3-4)**	651	17.3	224	36.3	194	29.2	233	0.0	0
	**MDR (5-6)**	1191	51.5	666	0.4	2	64.2	513	6.6	10
	**MDR (≥7)**	532	30.3	391	0.0	0	0.0	0	93.4	141
**ESBL/AmpC**	**Positive**	1311	27.9	360	16.3	87	89.7	717	97.4	147
**Stage**	**Slaughterhouse**	2524	95.9	1239	97.6	522	79.8	638	82.8	125
	**Retail**	253	4.1	53	2.4	13	20.2	161	17.2	26
**Sample type**	**Animal (caecum)**	2524	95.9	1239	97.6	522	79.8	638	82.8	125
	**Meat**	253	4.1	53	2.4	13	20.2	161	17.2	26
**Year**	**2014**	375	18.6	240	19.1	102	4.0	32	0.7	1
	**2015**	432	16.6	214	12.0	64	14.9	119	23.2	35
	**2016**	556	23.0	297	17.9	96	18.3	146	11.3	17
	**2017**	316	11.7	151	12.5	67	11.3	90	5.3	8
	**2018**	744	18.9	244	24.3	130	38.3	306	42.4	64
	**2019**	354	11.3	146	14.2	76	13.3	106	17.2	26
**Season**	**Spring**	379	16.3	210	15.0	80	9.8	78	7.3	11
	**Summer**	472	19.3	250	19.6	105	11.3	90	17.9	27
	**Autumn**	1380	44.2	571	49.0	262	56.9	455	60.9	92
	**Winter**	546	20.2	261	16.4	88	22.0	176	13.9	21

*Pan-S - Pan-susceptible; Mono - resistance to one antimicrobial class; Dual - resistance to 2 antimicrobial classes; MDR (3-4) - multi-resistance to 3 and/or 4 antimicrobial classes; MDR (5-6) - multi-resistance to 5 and/or 6 antimicrobial classes; MDR (>7) - multi-resistance to 7 or more antimicrobial classes.

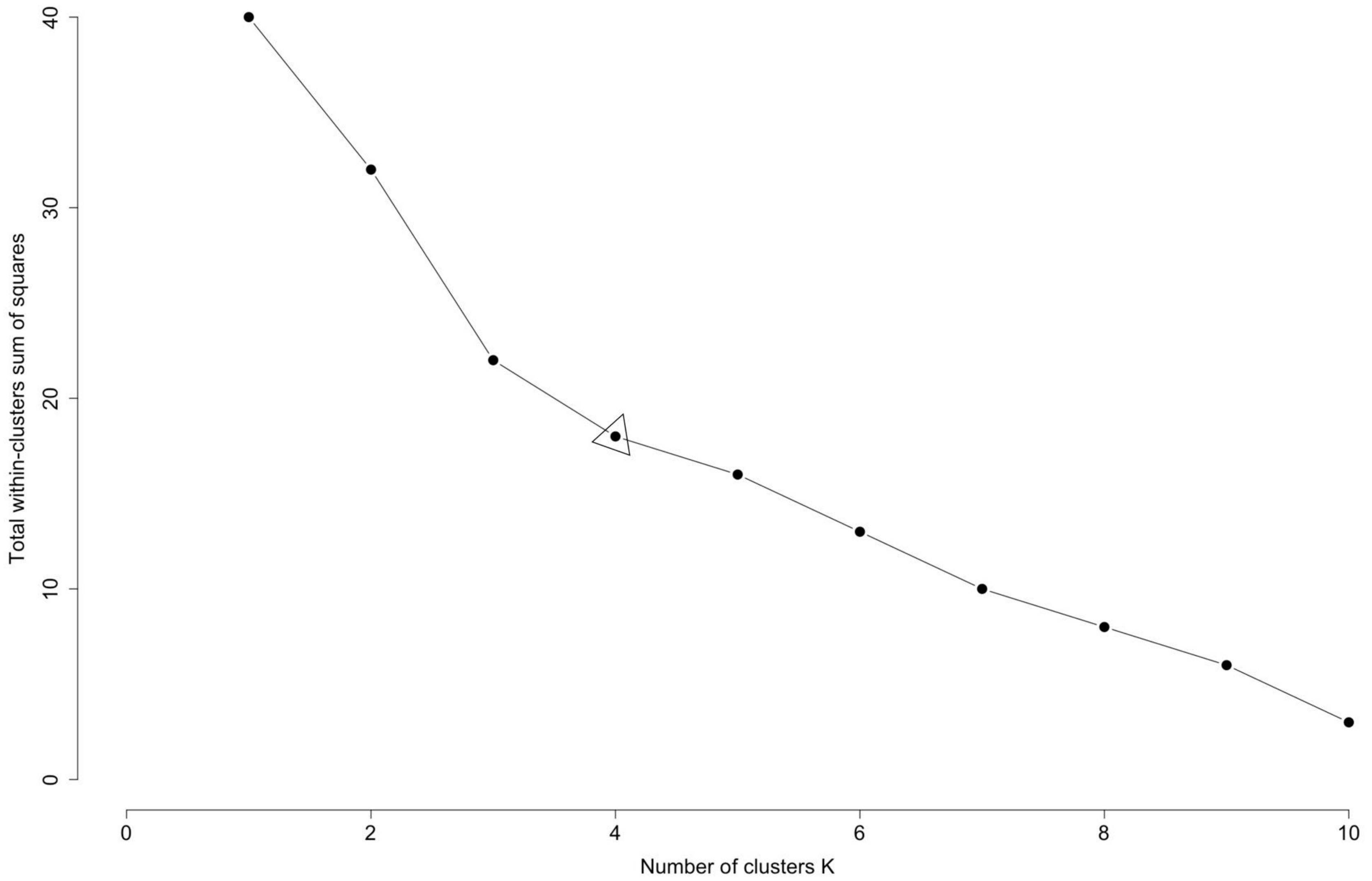

Figure S1: Number of optimal clusters for *E. coli* clustering model using the elbow method.

Table S2: Surveillance variables and resistance classification associated with clusters from *Salmonella* antimicrobial susceptibility testing results.

			Cluster 1 *n*= 141		Cluster 2 *n*= 619		Cluster 3 *n*= 30		Cluster 4 *n*= 129	
		**N**	**%**	**n**	**%**	**n**	**%**	**n**	**%**	**n**
**Population/ food**	**Broilers**	464	11.3	16	66.2	410	13.3	4	26.4	34
	**Broiler products**	206	21.3	30	22.8	141	6.7	2	25.6	33
	**Pork products**	249	67.4	95	11.0	68	80.0	24	48.1	62
**Resistance**	**Pan-S**	315	0.0	0	50.9	315	0.0	0	0.0	0
	**Mono**	290	0.0	0	42.3	262	93.3	28	0.0	0
	**Dual**	58	15.6	22	5.5	34	6.7	2	0.0	0
	**MDR (3-4)**	176	75.2	106	1.1	7	0.0	0	48.8	63
	**MDR (5-6)**	64	9.2	13	0.2	1	0.0	0	38.8	50
	**MDR (≥7)**	16	0.0	0	0.0	0	0.0	0	12.4	16
**Stage**	**Farm**	463	11.3	16	66.1	409	13.3	4	26.4	34
	**Slaughterhouse**	136	25.5	36	6.9	43	33.3	10	36.4	47
	**Processing plant**	312	61.7	87	26.3	163	50.0	15	36.4	47
	**Retail**	8	1.4	2	0.6	4	3.3	1	0.8	1
**Sample type**	**Environmental/Animal**	464	11.3	16	66.2	410	13.3	4	26.4	34
	**Carcase/neck skin**	229	33.3	47	18.3	113	36.7	11	45.0	58
	**Food**	226	55.3	78	15.5	96	50.0	15	28.7	37
**Year**	**2014**	105	13.5	19	11.5	71	6.7	2	10.1	13
	**2015**	390	24.1	34	48.3	299	33.3	10	36.4	47
	**2016**	130	25.5	36	11.1	69	13.3	4	16.3	21
	**2017**	118	12.1	17	12.9	80	23.3	7	10.9	14
	**2018**	119	8.5	12	13.6	84	13.3	4	14.7	19
	**2019**	57	16.3	23	2.6	16	10.0	3	11.6	15
**Season**	**Spring**	238	24.1	34	26.2	162	26.7	8	26.4	34
	**Summer**	285	20.6	29	33.0	204	20.0	6	35.7	46
	**Autumn**	275	39.0	55	28.4	176	30.0	9	27.1	35
	**Winter**	121	16.3	23	33.0	204	23.3	7	10.9	14

*Pan-S - Pan-susceptible; Mono - resistance to one antimicrobial classe; Dual - resistance to 2 antimicrobial classes; MDR (3-4) - multi-resistance to 3 and/or 4 antimicrobial classes; MDR (5-6) - multi-resistance to 5 and/or 6 antimicrobial classes; MDR (>7) - multi-resistance to 7 or more antimicrobial classes.

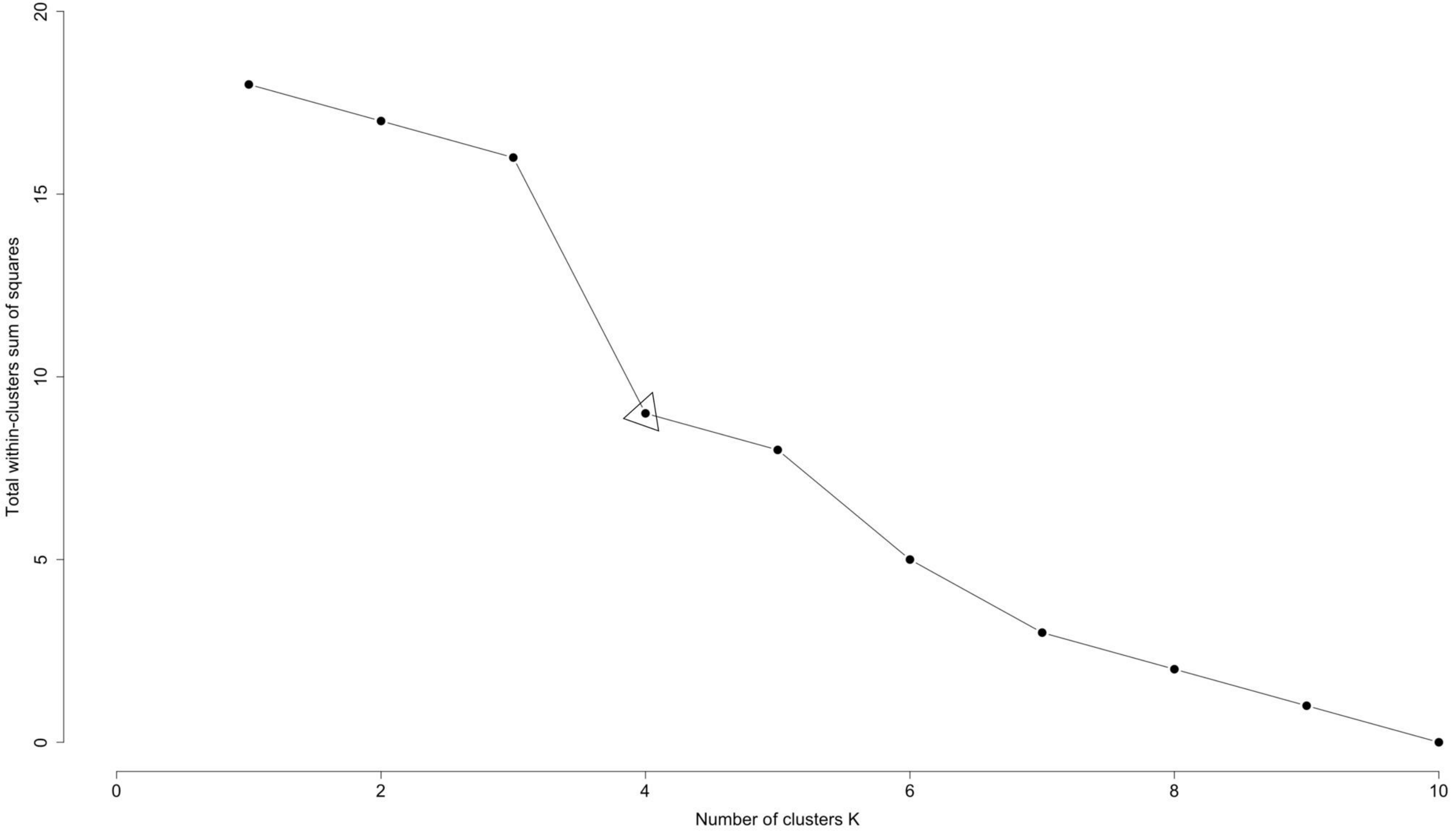

Figure S2: Number of optimal clusters for *Salmonella* clustering model using the elbow method.

Table S3: Surveillance variables and resistance classification associated with clusters from *Campylobacter* antimicrobial susceptibility testing results.

			**Cluster 1** *n*= 479		**Cluster 2** *n*= 11		**Cluster 3** *n*= 51	
		**N**	**%**	**n**	**%**	**n**	**%**	**n**
**Population/ food**	**Broilers**	360	65.8	315	72.7	8	72.5	37
	**Broiler products**	83	15.7	75	9.1	1	13.7	7
	**Turkeys**	98	18.6	89	18.2	2	13.7	7
**Resistance**	**Pan-S**	11	0.0	0	100.0	11	0.0	0
	**Mono**	60	2.5	12	0.0	0	94.1	48
	**Dual**	370	76.8	368	0.0	0	3.9	2
	**MDR (3-4)**	100	20.7	99	0.0	0	2.0	1
**Stage**	**Slaughterhouse**	458	84.3	404	90.9	10	86.3	44
	**Processing plant**	83	15.7	75	9.1	1	13.7	7
**Sample type**	**Animal (caecum)**	458	84.1	404	90.9	10	86.3	44
	**Food**	83	15.9	75	9.1	1	13.7	7
**Year**	**2014**	335	59.9	287	45.5	5	84.3	43
	**2015**	19	3.8	18	0.0	0	2.0	1
	**2016**	0	0.0	0	0.0	0	0.0	0
	**2017**	12	2.3	11	9.1	1	0.0	0
	**2018**	171	33.2	159	45.5	5	13.7	7
	**2019**	4	0.8	4	0.0	0	0.0	0
**Season**	**Spring**	82	14.2	68	9.1	1	25.5	13
	**Summer**	169	29.6	142	36.4	4	45.1	23
	**Autumn**	267	51.6	247	45.5	5	29.4	15
	**Winter**	23	4.6	22	9.1	1	0.0	0

*Pan-S - Pan-susceptible; Mono - resistance to one antimicrobial classe; Dual - resistance to 2 antimicrobial classes; MDR (3-4) - multi-resistance to 3 and/or 4 antimicrobial classes.

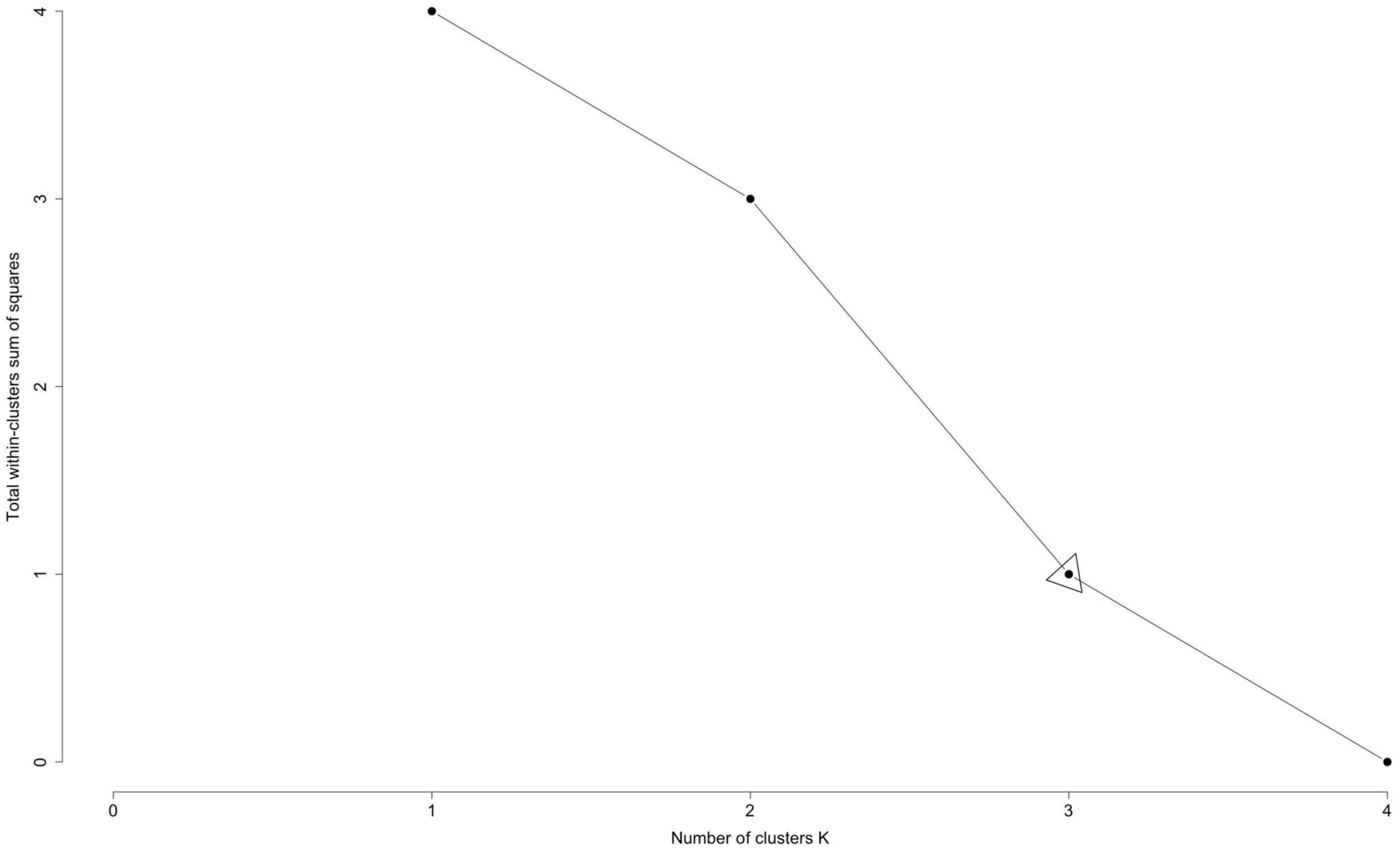

Figure S3: Number of optimal clusters for *Campylobacter* clustering model using the Elbow method.

Table S4: *Escherichia coli* β-lactam antimicrobial resistance and most frequent profiles in animal and food samples tested for ESBL/AmpC.

	β-lactam Antimicrobial Resistance				
	**Antimicrobial class**	**R (%)**	**N**		**Profile**
Food-producing animals *n* = 1092	C2G	19%	202	**1**	C3G-C4G (77%)
	C3G	98%	1067	**2**	C2G-C3G -C4G (8%)
	C4G	95%	1034	**3**	C2G-C3G-C4G-C3GCA (5%)
	C3G-CA	10%	105	**4**	C3G-C2G-C3GCA (3%)
	CBP	4%	43	**5**	C3G-C2G-C4G-CARB-C3GCA (2%)
	β-PEN	0.1%	1	**6**	C3G-C4G-CARB (1%)
Food *n* = 253	C2G	21%	52	**1**	C3G-C4G (74%)
	C3G	96%	244	**2**	C2G-C3G- C4G-C3GCA (8%)
	C4G	89%	225	**3**	C2G-C3G-C3GCA (6%)
	C3GCA	16%	41	**4**	C2G-C3G-C4G (4%)
	CBP	3%	7	**5**	C3G-C2G-C4G-CARB-C3GCA (2%)
	β-PEN	0%	0	**6**	C3G (1%)

*C2G/3G/4G - 2nd, 3rd and 4th generation cephalosporins, C3G-CA - 3rd generation cephalosporin and clavulanic acid; CBP - Carbapenems; β-PEN - β-lactam resistant penicillins
